# Heated Tobacco Products: Insights into Composition and Toxicity

**DOI:** 10.3390/toxics11080667

**Published:** 2023-08-02

**Authors:** Swapna Upadhyay, Mizanur Rahman, Gunnar Johanson, Lena Palmberg, Koustav Ganguly

**Affiliations:** Unit of Integrative Toxicology, Institute of Environmental Medicine, Karolinska Institutet, 171 77 Stockholm, Sweden; mizanur.rahman@ki.se (M.R.); gunnar.johanson@ki.se (G.J.); lena.palmberg@ki.se (L.P.)

**Keywords:** electronic cigarette, tobacco, smoking, heat not burn, IQOS

## Abstract

Heated tobacco products (HTPs) are novel products that allow users to inhale nicotine by heating (350 °C) reconstituted tobacco rather than combustion (900 °C) as in conventional cigarettes. HTP sticks containing reconstituted tobacco come in various flavours such as menthol, citrus, etc., like electronic cigarette liquids. Thus, the composition of HTP aerosol will also vary according to the flavouring agents added. Overall, the content of toxic chemicals in HTP aerosol appears to be lower than in cigarette smoke. However, the concentrations of more than twenty harmful and potentially harmful constituents have been reported to be higher in HTP aerosol than in cigarette smoke. Further, several toxic compounds not detected in cigarette smoke are also reported in HTP aerosol. Thus, the risks of HTP use remain unknown. Most of the available data on the composition and health effects of mainstream HTP aerosol exposure are generated by the tobacco industry. Few independent studies have reported short-term pathophysiological effects of HTP use. Currently available HTP toxicity data are mainly on the pulmonary and cardiovascular systems. Moreover, there are no long-term toxicity data and, therefore, the claims of the tobacco industry regarding HTPs as a safer alternative to traditional combustible cigarettes are unsubstantiated. Furthermore, HTP aerosol contains the highly addictive substance nicotine, which is harmful to the adolescent brain, developing foetuses, pregnant women, and also adults. Hence, comprehensive studies addressing the safety profiling related to long-term HTP use are warranted. With this background, the following review summarizes the current state of knowledge on HTP toxicity on four broad lines: composition of mainstream HTP aerosol compared to traditional combustible cigarette smoke, biomarkers of HTP exposure, health effects of HTP exposure, and the harm reduction aspect.

## 1. Introduction

Heated tobacco products (HTPs) are a new category of tobacco products with limited toxicological data. HTPs heat reconstituted tobacco to about 350 °C to generate nicotine-containing aerosol. This contrasts the burning of traditional combustible cigarettes (TCCs), where temperatures up to 900 °C are reached [1,2,3,4]. In some HTP products, temperatures of around 550 °C have been reported [1]. HTP products are currently available in more than 50 countries [1,2,3,4,5]. The total sale of HTP products is forecasted to reach nearly USD 68 billion by 2027, a seven-fold increase from 2020 [6]. HTP products are becoming increasingly popular among the youth (15–24 year), current smokers, and former smokers [7,8,9].

Table 1 lists some of the commonly available HTP products. Most of the HTP research data currently available were obtained for the tobacco heating system 2.2, which has been marketed as the “IQOS” (I Quit Ordinary Smoking) by Philip Morris International (PMI) [5]. The IQOS is operated by a battery-powered heating device with a heating blade that is inserted into a tobacco stick containing reconstituted tobacco (hereafter referred to as HTP sticks) [4]. HTP sticks come in different flavours (e.g., menthol, tobacco, chocolate, berry, citrus), as do electronic cigarette (ECIG) liquids. However, HTP devices heat reconstituted tobacco, whereas ECIGs heats a liquid that may or may not contain nicotine [1,2,3]. The products “iFuse”, “PloomTech”, “lil hybrid”, and “lil vapor” are hybrids between HTPs and ECIGs. Hybrid devices use technology like electronic nicotine and non-nicotine delivery systems to derive flavour elements from small amounts of tobacco. Here, the emission is passed over tobacco to heat it and pick up the flavour, and is eventually inhaled by the user [1].

Heating versus combustion is the basic operational difference between HTPs and TCCs. The tobacco industry claims that due to this operational difference, HTP aerosol contains fewer toxic chemicals compared to TCC smoke and, hence, it is a safer alternative to conventional smoking. Based on the current state of independent toxicological data and lack of long-term toxicity data on HTP, we consider these claims to be unsubstantiated. 

In this review article, we summarize the current state of knowledge on the chemical composition of mainstream HTP emissions compared to those of mainstream conventional cigarettes. Harmful and potentially harmful chemicals present in higher amounts in HTP emissions compared to TCCs that may result in previously unknown toxicological effects among users are highlighted. Further, an assessment of the biomarkers of exposure to HTP use during randomized clinical trials by the tobacco industry to demonstrate harm reduction is also discussed. A wide range of potential adverse health outcomes ranging from the pulmonary, cardiovascular, and immune modulatory effects of HTP use are reviewed. Finally, we discuss the potential of the harm reduction aspect of HTPs based on the current knowledge from ECIG users. This article is therefore divided into the following four sections: (I) composition of mainstream HTP aerosol compared to TCC smoke, (II) biomarkers of HTP exposure, (III) health effects of HTP exposure, and (IV) the harm reduction aspect.

## 2. Composition of Mainstream HTP Emissions

**Figure 1:**
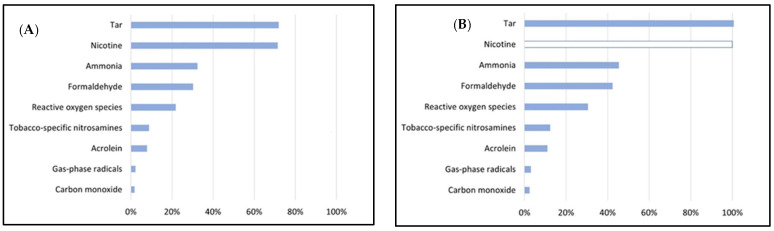
Graphical representation of the average amount of a few selected harmful and potentially harmful constituents in the mainstream heated tobacco product (**HTP**) emissions from: (**A**) one HTP stick relative to mainstream smoke from one traditional combustible cigarette. (**B**) Same as A, normalized for nicotine. Averages were calculated based on the midpoints (maximum − minimum/2) of each chemical shown in Table 2 and Table 3 [4,10,11,12,13,14,15].

This section compares the composition of mainstream HTP aerosol and mainstream cigarette smoke (Figure 1; Table 2 and Table 3) with respect to several harmful and potentially harmful constituents (HPHCs) including, e.g., nicotine, tobacco-specific nitrosamines (TSNAs), tar, carbon monoxide (CO), aromatic amines, hydrogen cyanide, ammonia, phenol, volatile organic compounds (VOCs), polycyclic aromatic hydrocarbons (PAHs), reactive oxygen species (ROS), and carbonyls [4,10,11,12,13,14,15]. The contents are given as mass per HTP stick or cigarette. The contents of ECIG aerosol are also given in cases where simultaneous measurements along with HTP aerosol and TCC aerosol have been reported under comparable puffing regimens [4,11,12,13]. Supplementary Tables S1–S4 list the specific details in relation to each study used for compiling the data on the composition of emissions in this review article regarding the smoking regimen, device, flavour, brand, etc.

The nicotine content in the tobacco fillers (per cigarette) is higher in the TCCs than the HTP sticks [4]. Similarly, the TSNA levels are also higher in TCCs. Correspondingly, the TSNA content in HTP aerosol is 7–17 times lower than TCC smoke [13]. The range of nicotine content in HTP aerosol (0.5–1.50 mg/cigarette) is lower compared to TCC smoke (0.7–2.1 mg/cigarette) (Table 3) [1]. However, some studies reported comparable nicotine content in HTP aerosol and TCC smoke [14]. Based on the surveyed literature [4,10,11,12,13,14,15], the range of total particulate matter in HTP aerosol is higher compared to TCC smoke. The percentage of free base is comparable between HTP aerosol and TCC smoke. The tar content is lower in the case of HTP aerosol compared to TCC smoke [1], although some reports suggest comparable tar emissions [14]. Propylene glycol and glycerin are detected in HTP aerosol. The CO levels in HTP aerosol is 40–60 times lower than in TCC smoke. Aromatic amines were not detected in HTP aerosol but were present in TCC smoke [14]. Total particulate and gas-phase radicals as well as reactive oxygen species are 40–80 and 1.5–8 times higher in TCC smoke compared to HTP aerosol, respectively (Table 2). The overall carbonyl content in TCC smoke is 3–16 times higher than in HTP aerosol (Table 2 and Table 3). Similarly, the levels of VOCs are also much higher in TCC smoke than in HTP aerosol (Table 2). Highly toxic formaldehyde cyanohydrin is also detected in HTP aerosol [16]. Almost three times higher levels of potentially carcinogenic acenapthene in HTP aerosol than in TCC smoke have also been reported [17]. Evidence of pyrolysis products has been reported in HTP aerosol following simulated experiments [14,16]. Analysis of the literature further shows the influence of puffing topography such as puff volume, puff duration, inter-puff interval (e.g., ISO—International Organization for Standardization-ISO 3308:2000, HCI—Health Canada Intense puffing regimens-T-115, CORESTA-Cooperation Centre for Scientific Research Relative to Tobacco-N°64) on the toxic emissions of HTP aerosol, ECIG aerosol, and TCC smoke [4,10,11,12,13,14,15,18]. The flavour (e.g., variations of menthol) and brand (University of Kentucky reference cigarette: 3R4F, 1R6F, etc.; commercially available: Marlboro Red 100, etc.) also influences the HTP, ECIG, and TCC emission constituents [4,10,11,12,13,14,15] (Supplementary Tables S1–S4). For example, the nicotine emission in the ISO regimen is 0.50 ± 0.03 mg per HTP stick, whereas that in the case of TCCs is 0.71 mg [14]. Correspondingly, for the HCI regime, the nicotine emission is 1.35 ± 0.07 mg per HTP stick, whereas the same for TCCs is 1.90 mg [14]. Similarly, for CORESTA, the nicotine emission per HTP stick is 1.46 ± 0.12 mg and the same for TCCs is 2.09 ± 0.09 mg and ECIGs is 1.56 ± 0.45 mg [4]. 

An overall description of the ISO, HCI, and CORESTA puffing regimens used in the different studies [4,10,11,12,13,14,15], based on which Figure 1 and Table 2 and Table 3 are prepared, are as follows:

- **ISO:** puff volume: 35 mL; duration: 2 s; interval: 60 s; six puffs per HTP stick; six puffs per TCC.
- **HCI:** puff volume: 55 mL; duration: 2 s; interval: 30 s; 12 puffs per HTP stick; 8–12 puffs per TCC; ECIG: 24–55 puffs.
- **CORESTA:** puff volume: 75 mL; duration: 2.5 s; interval: 30 s; 7–12 puffs per HTP stick; 11 puffs per TCC; ECIG: 10 puffs.

Specific details of the puffing regimens with reference to each study and corresponding emissions are provided in the Supplementary Tables S1–S4.

The current scientific literature on HTPs is dominated by tobacco-industry-sponsored research [5,19], warranting more independent studies on this topic. Independent examination of PMI’s IQOS emission data [20] on the US Food and Drug Administration’s HPHC list revealed 56 constituents to be higher in HTP aerosol (Marlboro HTP sticks) compared to TCC smoke (3R4F cigarettes). PMI reported levels of only 40 of 93 of these HPHCs on US Food and Drug Administration’s list. Among the not reported HPHCs, fifteen were two-fold or more and seven were more than ten-fold higher in HTP aerosol compared to TCC smoke [20] (Table 4). The above-reviewed data suggest HTP aerosol may expose users to lower levels of some toxicants than TCC smoke on one hand, and on the other hand, they also expose users to comparable as well as higher amounts of certain toxicants [19].

When comparing HTPs and TCCs, it should be kept in mind that HTP sticks are smaller (at least half the size) than TCCs, reflected as a lower nicotine content. The small size of the HTP sticks may lead to increased consumption, i.e., two sticks replacing one cigarette, to obtain a similar nicotine dose. The strive to reach a certain nicotine dose may explain why the widely popular IQOS device comes in two varieties, one where the battery discharges after approximately 6 min, allowing the use of one HTP stick in a smoking session, and one where the battery supports the use of two consecutive HTP sticks [21]. Thus, the technological evolution of HTP devices can also influence HTP aerosol exposure levels. As already discussed, the puffing pattern will also influence emission yields and exposure. 

In this context, it would plausibly be more appropriate to normalize the emissions from TCCs and HTPs with the mass of tobacco. However, most of the studies used for this review article lacked information on the mass of tobacco. Bekki et al. [10] reported the concentration of nicotine (mg/g) and TNSAs (ng/g) in tobacco fillers. The authors reported a similar concentration of nicotine (mg/g) in the fillers of both TCCs and HTPs (TCC: 3R4F: 19.7 ± 0.2, 1R5F: 15.9 ± 0.3; IQOS: regular: 15.7 ± 0.2; menthol: 17.1 ± 0.6) [10]. The nicotine in the mainstream smoke (per cigarette) was also comparable (Supplementary Table S1) between the 1R5F, IQOS regular, and IQOS menthol (1.0–1.2 mg/cigarette) and relatively higher in the 3R4F (1.7 mg/cigarette) [10]. Based on the above values, the authors further estimated the transfer rates of nicotine at 23.4% (IQOS regular) and 23.5 (IQOS menthol), whereas the same for TCC-3R4F was 11.3% and TCC-1R5F was 11.5% [10]. This indicates that IQOS has a more effective nicotine transfer rate than TCCs [10].

## 3. Biomarkers of Exposure

**Figure 2:**
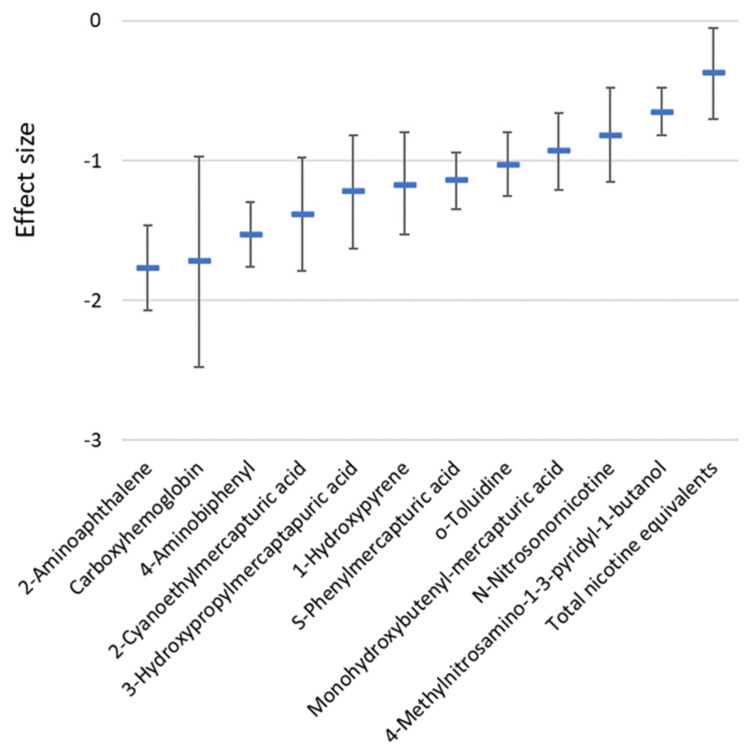
Biomarkers of exposure effect sizes (expressed relative to the variation) and their 95% confidence interval in a group of heated tobacco product users versus a group of traditional combustible cigarette smokers (data from Drovandi et al., 2020 [22], Table 2).

A systematic review and meta-analysis study by Drovandi et al. [22] evaluated 12 commonly reported biomarkers of exposure among 1766 subjects involving 10 non- blinded randomized controlled trials comparing TCC smokers and HTP users revealed lower levels of all the twelve biomarkers among the latter. All the ten eligible studies used for the meta-analysis were funded by the tobacco industry. The measured biomarkers were 1-hydroxypyrene, 2-aminoaphthalene (2-AN), 3-cyanoethylmercapturic acid (CEMA), 3-hydroxypropylmercaptauric acid (3-HPMA), 4-aminobiphenyl (4-ABP), 4-(methylnitrosamino)-1-(3-pyridyl)-1-butanol (NNAL), carboxyhemoglobin (COHb), monohydroxybutenyl-mercapturic acid, n-nitrosonornicotine (NNN), o-toluidine, phenylmercapturic acid, and total nicotine equivalents (TNeq). The reductions were most prominent for COHb, 2-AN, 4-ABP, and CEMA. Figure 2 shows the biomarker effect sizes for the HTP group versus the TCC group. Compared to smoking abstinence, eight biomarkers were unaffected among HTP users, while four (3-HPMA, NNN, NNAL, and TNeq) were significantly elevated [22] (Table 5). The findings that the levels of all 12 evaluated biomarkers of exposure were significantly lower among the HTP users compared to the TCC users need to be interpreted with caution. First, there were no independent studies in the analysis, and second, the number of studies included is small [22]. Further, the HTP devices used for the 10 studies were mixed (IQOS, Glo, and precursors) [22]. Hence, the exposures may be regarded as inconsistent and variable. Therefore, the safety claims of the tobacco industry based on the reduced levels of these 12 biomarkers of exposure is unsubstantiated. On the other hand, the findings that the levels of 8 of the 12 biomarkers of exposure among HTP participants were not significantly different compared to the smoking abstinence group, together with the established fact that HTP emissions contain a wide range of toxic compounds, clearly demonstrate that HTP use is unsafe. To summarize, considering the small number of studies included and the limited range of biomarkers of exposure assessed further independent research on the safety of HTP use is warranted [22].

## 4. Health Effects of Exposure to Mainstream HTP Emissions

The current knowledge on the plausible adverse health outcomes of exposure to HTP aerosol is primarily focused on the pulmonary and cardiovascular systems. However, there is a paucity of long-term toxicity data on the use of HTP products.

### 4.1. Pulmonary Effects

Independent analysis of publicly available tobacco industry data revealed a significant reduction in white blood cells among adult smokers (Japan-based study) when they switched to HTPs (n = 148; inclusion criteria: asymptomatic with at least 10 cigarettes/day for 3 years). However, no improvement in pulmonary function (forced expiratory volume in 1 s) or reduction in the systemic acute phase inflammatory marker C-reactive protein (CRP) were observed [23,24]. A three-year follow-up study among smokers with chronic obstructive pulmonary disease (COPD) (n = 38 subjects; age-and sex-matched) who abstained from smoking or switched to HTPs observed consistent improvement in pulmonary function, exercise tolerance (6 min walk distance), quality of life (COPD assessment test score), and rate of disease exacerbations (GOLD—Global Initiative for Chronic Obstructive Lung Disease grading) compared to COPD patients who continued smoking [25]. Another study (50 male subjects) evaluating the acute pulmonary effects of HTP aerosol exposure detected significantly increased exhaled CO and airway resistance (respiratory impedance and respiratory resistance). Significantly decreased oxygen saturation, forced expiratory flow at 25% and 50% of vital capacity, peak expiratory flow, and diffusion lung capacity for CO were detected [26]. The changes detected were not at magnitudes of immediate clinical importance but enough to raise concern about the safety regarding the long-term use of HTP products. Two case reports of HTP-use-associated acute eosinophilic pneumonia have been reported. In both these case reports, the subjects were males (age: 16 and 20 years) who were not cigarette smokers and started using HTP tobacco products 2 weeks and 6 months (20 HTP sticks/day and doubled the consumption in last 2 weeks), respectively, prior to hospitalization [27,28,29].

Inhalation exposure of HTP aerosol in female rats for 90 days (6 h/day; targeted nicotine dose: 23 µg/L of aerosol; 7–10 rats/group) resulted in significantly increased lung weight, bronchoalveolar lavage inflammatory cell recruitment, epithelial hyperplasia and metaplasia, and elevated levels of bronchoalveolar lavage inflammatory markers (monocyte chemoattractant protein, macrophage inflammatory protein, myeloperoxidase, plasminogen activator inhibitor) in the airways compared to controls. However, the inflammatory response was significantly lower when compared to female rats exposed to TCC smoke [23,24]. Short-term (5 h/day for 14 days; HCI puffing regimen) inhalation exposure to HTP aerosol in mice resulted in increased albumin in the bronchoalveolar lavage fluid, indicating lung epithelial barrier leakage and infiltration of leukocytes in the lungs. Increased numbers of cluster of differentiation 4 (CD4)+ and interleukin (IL)-17A+ T cells (marker of T cell immune response) as well as increased numbers of CD4+ and RAR-related orphan receptor gamma-t+ T cells (an inflammatory T cell subtype expressing the transcription factor essential for promoting differentiation into pro-inflammatory Th17 cells) and elevated levels of several proinflammatory cytokines (chemokine ligand-4, 5, 11; chemokine (C-X-C motif) ligand 1; IL-2, IL-5, IL-9, IL-13; interferon gamma; and tumour necrosis factor alpha) in the bronchoalveolar lavage fluid compared to sham-exposed controls were detected [30].

Several studies using human bronchial epithelial cells demonstrated that HTP exposure resulted in oxidative stress and pro-inflammatory response [23,31]. Higher cytotoxicity was also reported in human bronchial epithelial cells following HTP exposure compared to corresponding sham and ECIG exposure [23,30,31,32]. These observations are attributable to the presence of tar, VOCs, PAHs, carbonyl compounds, and other HPHCs in HTP aerosol, resulting in increased free radical exposure [31]. Altered mitochondrial respiration has also been reported following exposure to HTP in human bronchial epithelial cells and airway smooth muscle cells [33,34]. In a recent study, Rahman et al. (2022) [35] evaluated the pulmonary molecular toxicity of HTP aerosol (flavour: intense menthol with citrus) using physiologically relevant bronchial and alveolar lung mucosa models developed at the air–liquid interface. Six puffing sessions with either HTP aerosol or sham (clean air) were conducted with 1 h intervals during a one-day exposure experiment. Each exposure session consisted of 10 puffs according to the non-intense ISO puffing regimen (frequency: one puff per minute, volume: 35 mL, duration: 2 s). The bronchial model used in this study was developed using primary bronchial epithelial cells and the alveolar model using representative the human type II pneumocyte (NCI-H441 from ATCC HTB-174) cell line. Elevated levels of total cellular ROS, stress-responsive nuclear factor kappa-B, and DNA damage markers (8-hydroxy-2′-deoxyguanosine/8-OHdG, phosphorylated histone H2AX, cleaved poly-(ADP-Ribose) polymerase/PARP) were detected in HTP-aerosol-exposed bronchial models [35]. In the case of the alveolar model, only increased total cellular ROS, 8-OHdG, and PARP were detected on HTP aerosol exposure. Transcriptomic analysis revealed enrichment of oestrogen biosynthesis, ferroptosis, superoxide radical degradation, xenobiotics, and α-tocopherol degradation pathways in both the bronchial and alveolar models due to HTP aerosol exposure [35]. Increased lipid peroxidation was detected in both HTP-aerosol-exposed bronchial and alveolar models, which was inhibited by ferrostatin-1(lipid peroxidation and ferroptosis inhibitor) [35]. The findings of this study therefore indicated oxidative stress, DNA damage, lipid peroxidation, and ferroptosis as key events of HTP toxicity, consistent with the known mechanisms of toxicity for TCC.

### 4.2. Cardiovascular Effects

The effects of exposure to mainstream HTP emissions are regarded as potentially harmful to cardiovascular health though less than that of TCC smoke [36]. Acute effects on heart rate, blood pressure, and arterial stiffness have been reported among HTP users that are similar to TCC smokers [36]. One study involving 22 current TCC smokers with no comorbidities exhibited similar acute effects of HTP products and TCCs based on the evaluation of a panel of cardiovascular assessments: heart rate, blood pressure, carotid–femoral pulse wave velocity, and brachial–ankle pulse wave velocity, which were significantly elevated from baseline [36,37]. Another study including twenty active, healthy smokers (10 men and 10 women) were studied to assess the acute effect of HTP use on arterial stiffness in comparison to ECIG use and TCC smoking. Peripheral systolic blood pressure and mean arterial pressure increased significantly within the TCC, ECIG, and HTP groups by more than 3% compared to before exposure. Heart rate increased by more than 9% in the TCC and HTP groups. The augmentation index, a measure of arterial stiffness, was also significantly increased in the HTP group after 5 min, whereas in the TCC group, increased phenomena were observed till 15 min after smoking. The pulse wave velocity parameter, another measure of arterial stiffness, only showed a trend of alteration in the HTP and ECIG group and was significantly altered in the TCC group 15 min after exposure [38].

A study in rats indicated that acute exposure to HTP aerosol impairs arterial flow-mediated dilation. Other studies also demonstrated that HTP aerosol can rapidly and substantially impair endothelial function comparably to TCC smoke. The post-exposure serum nicotine levels were more than 4-fold higher in the HTP-aerosol-exposed rats compared to those exposed to TCC smoke [39]. An independent evaluation of tobacco-industry-sponsored research data from a Japanese cohort (70 HTP users, 41 TCC smokers, and 37 smoking abstainers) identified significant improvement of only 4 out of 13 systemic biomarkers of harm. In an American cohort (47 HTP users, 32 TCC smokers, and 9 smoking abstainers), there was significant improvement of only one out of 24 measured systemic biomarkers of harm. Systemic biomarkers exhibiting significant improvement among individuals switching to HTPs from TCCs included an increase in high-density lipoprotein and reduction in inflammatory indicators (white blood cell count, soluble intercellular adhesion molecule 1, and prostaglandin F2 alpha) [19].

### 4.3. Other Effects

Data from the tobacco industry indicate immunomodulatory effects of HTP aerosol exposure. Rats (7–10 per group) exposed to HTP aerosol developed 270% and 75% higher systemic neutrophilia compared to sham- and TCC-smoke-exposed rats. In female rats, following a 6-week recovery period, the blood neutrophil counts remained elevated compared to both sham- and TCC-exposed-animals. HTP-exposed rats also exhibited higher levels of thymic atrophy (by gross organ weight and histology) than both the sham- and TCC-smoke-exposed groups [24]. An independent examination of tobacco industry data further revealed significant increases in alanine amino transferase activity, liver weight, and hepatocellular vascularization (in females only) following 90 days of exposure to HTP aerosol of male and female rats compared to sham exposure, indicating plausible hepatoxicity [40,41]. Discoloration in the enamel, dentin, and composite resin restorations from baseline was observed following 3 weeks of HTP aerosol exposure, though to a significantly lesser extent compared to TCC smoke exposure in tobacco-industry-sponsored research [42,43].

### 4.4. Harm Reduction

One of the main claims by the tobacco industry regarding HTP is harm reduction although such claims remain unsubstantiated [44]. ECIG was also launched on the market with similar claims. In addition to the outbreak of e-cigarette, or vaping, product-use-associated lung injury (EVALI) [45] in the US, there are several chronic and long-term effects of ECIG use that raise concerns [46]. EVALI peaked during autumn 2019, resulting in the hospitalization and death of nearly 2800 subjects [45], where most of the affected individuals were in their youth. Sixty-eight deaths [45] have been confirmed to be due to EVALI up till February 2020. Information about ECIG usage can be extrapolated for HTPs as well to assess the risks. 

The role of ECIGs to help quit smoking or to reduce harm compared to TCCs remains unsubstantiated even though ECIGs were launched on the market much earlier than the modern HTPs. Most ECIG liquids with extremely attractive flavours previously contained nicotine. Due to recent amendments of tobacco directives globally, nicotine is sold separately, and both ECIG liquid and nicotine are sold only to adults (≥18 years). It is important to note here that individuals at 18 years old are still in their youth. Though such regulations can somewhat reduce children’s access to ECIGs, they cannot substantially inhibit it due to access through other channels in society. Similar risks remain with HTPs as well. Thus, HTPs, similar to ECIGs, can act as a gateway to conventional smoking and the use of other tobacco products like snus or waterpipes [47,48]. Risks that some of these users may be exposed to drugs (such as marijuana) and eventually become addicted cannot be ruled out.

ECIGs, instead of helping as a tool for quitting smoking, have eventually led to increased dual use (ECIGs and TCCs). The Population Assessment of Tobacco and Health study reported that 37.4% of adults and 43% of youths used multiple nicotine products, the most common combination being TCCs and ECIGs [49]. Other surveys among youths in the US [50] and Poland [51] have shown comparably high prevalence of dual use, with more than half of all ECIG users concomitantly smoking conventional cigarettes. A study from Germany [52] reported the prevalence of dual use to be around 50%. The findings of the study from Poland [51] suggested that adolescent dual users are more addicted to nicotine in view of their smoking behaviour (e.g., time to first cigarette, smoking intensity). These findings were also supported by the study from Germany [52], in which adult dual users had significantly higher scores in the Fagerstrom test for nicotine dependence. 

Furthermore, it has also been reported that dual users had the highest urine cotinine concentration. Cotinine is the predominant metabolite of nicotine. Measuring cotinine is preferred to measuring nicotine because cotinine remains in the body longer and correlates well to the exposure to nicotine for both smokers and nonsmokers exposed to environmental tobacco smoke [53]. A Korean study [54] (n = 7505 and ≥19 years) reported that about 85% of male ECIG users were, in fact, dual users. The dual users had greater nicotine dependence and higher urinary cotinine levels compared to only conventional cigarette smokers. Park et al. [55] also found that dual users had a higher urine cotinine concentration than that of the TCC smokers, and that the majority of ECIG users were dual users.

In a cross-sectional study, Osei et al. [56] found that dual use of ECIGs and TCCs was associated with significantly higher odds of cardiovascular disease compared with current TCC users who never used ECIGs (odds ratio 1.36; 95% confidence interval, 1.18–1.56). Furthermore, there appears to be no significant association between ECIG use and cardiovascular disease among people who never smoke TCCs, indicating a synergistic effect in dual users. Another study [57] reported that dual users had increased odds (OR 4.02, 95% CI 1.48–10.93) of metabolic syndrome among females, with increasing OR with increasing age. This indicates-gender specific effects among dual users. Hedman et al. [48] showed that all respiratory symptoms were more common among dual users in their cross-sectional study in northern Sweden [47]. Furthermore, dual users showed the highest association with self-reported COPD diagnosis (adjusted OR 4.39 (3.98, 4.85)) [58]. They also found that vaping was significantly associated with self-reported COPD diagnosis in adults, even among vapers who never smoked [58]. 

## 5. Conclusions

To summarize, HTPs are novel products that allow the user to inhale nicotine by heating tobacco rather than combustion as in conventional cigarettes. However, the presence of CO and a number of other TCC smoke constituents in HTP aerosol may correspond to what would be expected from slight combustion. HTP sticks have a smaller size and lower amount of tobacco than TCCs. Therefore, the amount of nicotine delivered in the mainstream emission from an HTP stick is generally lower than that delivered in the mainstream smoke from a TCC. However, technological advancements of HTP devices, such as improvement in the battery capacity, may result in increased rather than reduced consumption, e.g., by shifting from one to two sticks per smoking session. Apart from nicotine and CO, several other chemicals are also present in HTP aerosol, including a number of so-called HPHCs. The levels of these HPHCs are influenced by several factors such as the puffing regime, the HTP device, and additives to the HTP stick. Several HPHCs have been detected in higher concentrations in HTP aerosol than in TCC smoke. Currently, there is not sufficient evidence from independent sources to draw conclusions on the health effects of long-term use of HTP sticks. Few independent studies have reported short-term pathophysiological effects of HTP use. 

Multiple tobacco-industry-sponsored studies on human bronchial epithelial cells, coronary arterial endothelial cells, 3D nasal culture models, gingival epithelial organotypic cultures, monocytic cells, and mouse models indicate lower toxicity of HTP aerosol compared to TCC smoke [41]. In general, tobacco-industry-sponsored research highlights the findings of HTP emission exposure mainly in comparison to TCC smoke. However, it is important to note that potential adverse effects have been observed while comparing HTP-emission-exposed groups to unexposed groups. Thus, comprehensive studies addressing the safety profiling related to long-term HTP use are required. 

Nevertheless, it must be noted that HTP aerosol does contain nicotine, allowing users to inhale nicotine. Nicotine is highly addictive, and exposure to nicotine is harmful to the adolescent brain (the brain develops until about 25 years of age), particularly in the regions controlling attention, learning, mood, and impulse control. The use of nicotine among adolescents may also act as a gateway for future use and addiction to other drugs. Nicotine is toxic to developing foetuses and harmful for pregnant women. The use of nicotine is also harmful for adults [2,3]. Therefore, more independent and long-term research is necessary for evaluating the safety of HTP tobacco products.

In conclusion, the health effects of short-term and, in particular, long-term use of HTP sticks are uncertain. Accordingly, the European Respiratory Society recommended against the use of any product, including HTP tobacco products, that can potentially damage the lungs and human health [44]. It can be predicted that similar trends will follow in the case of HTPs to those observed for ECIGs, particularly regarding the dual usage resulting in higher nicotine intake. Furthermore, if the molecular mechanisms are considered, HTPs and TCCs share similar adverse outcome pathways including oxidative stress, inflammation, and lipid peroxidation, as well as the newly identified ferroptosis. Clearly, exposure to nicotine and HTPs serving as a gateway for nicotine addiction among adolescents and youths remains the foremost risk. This not only endangers the downward trend of tobacco usage observed particularly in developed nations in recent years but also threatens the implementation of the United Nations agenda 2030 goal 3 to ensure healthy lives and promote well-being for all at all ages. One of the means of implementation for the goal 3 targets is tobacco control. Further, it remains to be addressed how the HPHCs not present in TCCs but present in HTPs might cause harm among HTP users. Hence, one can reasonably anticipate the emergence of unknown modes of toxicity due to HTP use.

## Figures and Tables

**Table 1:** Examples of some HTP tobacco products [1,2,3,4,5]. Hybrid is a combination of features of HTP products and electronic cigarettes.

Product Name	Company	Type
Accord	Philip Morris International	HTP
Eclipse	RJ Reynolds Tobacco Company	HTP
iFuse	British American Tobacco	Hybrid
IQOS	Philip Morris International	HTP
Kent Glo	British American Tobacco	HTP
lil hybrid, lil vapor	The Korea Tobacco and Ginseng Corporation	Hybrid
lil plus, lil mini	The Korea Tobacco and Ginseng Corporation	HTP
PloomTech	Japan Tobacco International	Hybrid
Premier	RJ Reynolds Tobacco Company	HTP

**Table 2:** Comparative range of harmful and potentially harmful constituents (HPHCs; per stick or cigarette) present in the heated tobacco product mainstream aerosol (HTP aerosol) and mainstream smoke of traditional combustible cigarettes (TCC smoke) [4,10,11,12,13,14,15]. Blanks (-) indicate not reported/not quantified/below level of detection. Single values are provided where ranges could not be obtained. ISO, HCI, and CORESTA puffing regimens were used. Reported HTP aerosol data were obtained from tobacco- and menthol-flavoured HTP sticks. TCC smoke data were obtained from reference-grade (3R4F, 1R6F) and Marlboro Red 100 cigarettes.

(Per Cigarette)	HTP Stick	TCC Cigarette
Nicotine (mg)	4.7–5.1	8.7–15.0
Tobacco-specific nitrosamines (TSNAs) (ng)		
N-Nitrosonornicotine (NNN)	94.4–101.0	1899.0–1691.0
N’-Nitrosoanatabine (NAT)	94.5–99.8	913.0–1341.0
N-Nitrosoanabasine (NAB)	2.6–5.6	46.0–65.0
Nicotine-derived nitrosoamine ketone (NNK)	51.1–58.2	412.0–532.0
	**HTP Aerosol**	**TCC Smoke**
Total particulate matter (mg)	12.9–55.8	9.8–37.7
% Free base	5.7–13.6	5.8–14.5
Tar (mg)	7.5–16.6	8.0–25.50
Propylene glycol (mg)	0.2–0.6	-
Glycerin (mg)	1.6–3.8	0.80–2.3
Nicotine (mg)	0.5–1.5	0.7–2.1
Carbon monoxide (mg)	0.3–0.5	11.2–33.0
TSNAs (ng)		
NNN	5.00–24.9	92.1–311.1
NAT	6.1–37.2	92.9–246.4
NAB	2.6–5.5	9.60–30.4
NNK	3.5–13.8	85.50–250.4
Aromatic amines (ng)		
1-Aminonaphthalene	-	10.6–21.6
2-Aminonaphthalene	-	5.7–10.1
3-Aminobiphenyl	-	2.0–4.2
4-Aminobiphenyl	-	1.0–2.2
Hydrogen cyanide (µg)	-	70.9–319.0
Ammonia (µg)	2.4–10.5	11.1–28.7
Phenol (µg)	1.2	7.0–14.8
Polycyclic aromatic hydrocarbon (PAH) Benzo(a)pyrene (ng)	-	6.7–16.2
Reactive oxygen species (nmol H_2_O_2_)	6.3	10.7–46.8
Gas phase	1.9	2.3–2210
Particle phase	4.3	7.8–24.7
Particulate-phase radicals (pmol)	-	79.4
Volatile organic compounds (µg)		
1,3-Butadiene	0.5	38.5–76.5
Isoprene	0.6–3.0	395.0–863.0
Acrylonitrile	0.2	26.4–67.0
Benzene	0.1 –0.6	47.7–104.0
Toluene	0.8–2.5	73.6–208.0

**Table 3:** Comparative range of harmful and potentially harmful constituents (HPHCs, per cigarette) present in the heated tobacco product mainstream aerosol (HTP aerosol), mainstream smoke of traditional combustible cigarettes (TCC smoke), and electronic cigarette mainstream aerosol (ECIG aerosol) [4,10,11,12,13,14,15]. Blanks (-) indicate not reported/not quantified/below level of detection. Single values are provided where ranges could not be obtained. ISO, HCI, and CORESTA puffing regimens were used. Reported HTP aerosol data were obtained from tobacco- and menthol-flavoured HTP sticks, TCC smoke data were obtained from reference-grade (3R4F, 1R6F) and Marlboro Red 100 cigarettes, ECIG aerosol data were obtained from tobacco-flavoured ECIG liquid using 1st- and 2nd-generation ECIG devices at 10 and 14 wattage.

HPHC	HTP Aerosol	TCC Smoke	ECIG Aerosol
Nicotine (mg)	0.5–1.5	0.7–2.1	0.07–1.73
Total gas-phase radicals (pmol)	12.5–12.6	567.6	5.3–47.8
Non-polar	13.9–14.3	449.9	2.4–19.2
Polar	6.8–8.2	9.6	5.9–43.3
Carbonyls (µg)			
Formaldehyde	0.9–22.6	3.2–74.4	0.5–3.7
Acetaldehyde	128.5–301. 5	567.0–1534	0.8–2.9
Acetone	18.8–48.37	210–775.6	-
Acrolein	4.0–13.1	56.7–160.9	0.3–1.1
Propionaldehyde	9.6–22.3	48.4–124.0	-
Crotonaldehyde	1.4–6.4	10.10–65.7	-
Methacrolein	6.5	85.5	-
Butyraldehyde	14.9–30.7	22.2–65.0	-
Valeraldehyde	20.1	-	-
Glyoxal	3.1	-	-
Methyl glyoxal	33.5	-	-
2-Butanone	4.2–6.5	11.0–220.5	-

**Table 4:** List of harmful and potentially harmful constituents (HPHCs) reported to be more than two-fold higher in heated tobacco product mainstream aerosol (HTP aerosol) compared to mainstream smoke of traditional combustible cigarettes (TCC smoke) (adapted from St Helen et al., 2018) [20].

	HPHC	Fold Increase in HTP Aerosol Compared to TCC Smoke
1	1,4-Dioxane, 2-ethyl-5-methyl-	137
2	Hexadecanoic acid, ethyl ester	60
3	Trans-4-hydroxymethyl-2-methyl-1,3-dioxolane	47
4	Stearate, ethyl-	24
5	12,14-Labdadiene-7,8-diol, (8a,12E)	21
6	Butylated hydroxytoluene	18
7	Ethyl linoleate	16
8	Labdane-8,15-diol, (13S)	9
9	Propylene glycol	6
10	2-Furanmethanol	4
11	Butyrolactone	5
12	Methyl furoate	4
13	2-Cyclopentene-1,4-dione	4
14	2-Furanmethanol, 5-methyl-	3
15	Ethyl linolenate	3
16	2-Methylcyclobutane-1,3-dione	3
17	Lanost-8-en-3-ol, 24-methylene-, (3beta)	3
18	2-Furancarboxaldehyde, 5-methyl-	3
19	Eicosane, 2-methyl-	3
20	1,2,3-Propanetriol, diacetate (diacetin)	2
21	Glycidol	2
22	Heneicosane, 2-methyl-	2

**Table 5:** Significantly elevated biomarkers of exposure effect sizes (expressed relative to the variance) and their 95% confidence intervals for heated tobacco product users compared to smoking abstinence groups (data from Drovandi et al., 2020 [22], Table 2).

Biomarkers of Exposure	Effect Size	*p*-Value
3-Hydroxypropylmercaptauric acid (3-HPMA)	0.21 (0.02, 0.40)	0.027
4-(Methylnitrosamino)-1-(3-pyridyl)-1-butanol (NNAL)	0.11 (0.03, 0.18)	0.005
N-Nitrosonornicotine (NNN)	0.22 (0.01, 0.43)	0.041
Total nicotine equivalents (TNeq)	1.91 (1.40, 2.41)	<0.001

## Data Availability

Not applicable.

## Supplementary Materials

The following supporting information can be downloaded at: https://www.mdpi.com/article/10.3390/toxics11080667/s1, Supplementary Table S1: Concentration of harmful and potentially harmful constituents (per cigarette) present in heated tobacco product mainstream aerosol (HTP aerosol) and mainstream smoke of traditional combustible cigarettes (TCC smoke); Supplementary Table S2: Nicotine concentration present in heated tobacco product mainstream aerosol (HTP aerosol), mainstream smoke of traditional combustible cigarettes (TCC smoke), and mainstream electronic cigarette aerosol (ECIG aerosol); Supplementary Table S3: Comparative range of harmful and potentially harmful constituents (per cigarette) present in heated tobacco product mainstream aerosol (HTP aerosol) and mainstream smoke of traditional combustible cigarettes (TCC smoke); Supplementary Table S4: Comparative range of harmful and potentially harmful constituents (per cigarette) present in heated tobacco product mainstream aerosol (HTP aerosol), mainstream smoke of traditional combustible cigarettes (TCC smoke), and mainstream electronic cigarette aerosol (ECIG aerosol).
