# Supplementary material for: Heated Tobacco Products: Insights into Composition and Toxicity
Source: Toxics. 2023 Aug 2;11(8):667. doi: 10.3390/toxics11080667 (PMC10459283; doi:10.3390/toxics11080667)
Supplement: Supplementary file 1 [file toxics-11-00667-s001.zip › toxics-2327771-supplementary.pdf]

Supplementary Section

Heated Tobacco products: Insights into composition and toxicity.

Swapna Upadhyay[1*], Mizanur Rahman[1], Gunnar Johanson[1], Lena Palmberg[1], Koustav Ganguly[1*]

[1]Unit of Integrative Toxicology, Institute of Environmental Medicine, Karolinska Institutet, Stockholm, 171 77, Sweden.

Name	Email	ORCID ID
Swapna Upadhyay	swapna.upadhyay@ki.se	0000-0003-4699-4082
Mizanur Rahman	mizanur.rahman@ki.se	0000-0003-4129-9822
Gunnar Johanson	gunnar.johanson@ki.se	0000-0002-8759-9567
Lena Palmberg	lena.palmberg@ki.se	0000-0001-5650-4484
Koustav Ganguly	koustav.ganguly@ki.se	0000-0001-8531-8154

All authors contributed equally.

*Correspondence

Koustav Ganguly (koustav.ganguly@ki.se)

Unit of Integrative Toxicology, Institute of Environmental Medicine, Karolinska Institutet, Stockholm, 171 77, Sweden

Swapna Upadhyay (swapna.upadhyay@ki.se)

Unit of Integrative Toxicology, Institute of Environmental Medicine, Karolinska Institutet, Stockholm, 171 77, Sweden

Financial support: Swedish Public Health Agency and Swedish Heart Lung Foundation

Supplementary table S1: Concentration of harmful and potentially harmful constituents (per cigarette) present in heated tobacco product mainstream aerosol (HTP aerosol) and mainstream smoke of traditional combustible cigarettes (TCC smoke). Reported HTP aerosol data was obtained from tobacco/regular and menthol flavoured HTP sticks, TCC smoke data was obtained from reference grade (3R4F, 1R5F) cigarettes. Values are mean (standard deviation). Source: Bekki et al. 2017 [1].

	**HTP sticks**	**TCC cigarette**
Nicotine (mg)	4.7 (0.1) - regular<br>5.1 (0.2) - menthol	15.0 (0.1) - 3R4F<br>8.7 (0.1) -1R5F
Tobacco specific nitrosamines (TSNAs)		
N-nitrosonornicotine (NNN) (ng)	94.4(1.4) - regular<br>101.0 (2.8) - menthol	1899.0 (66.0) - 3R4F<br>1691.0 (67.0) - 1R5F
N´-nitrosoanatabine (NAT) (ng)	99.8 (1.46) - regular<br>94.5 (2.0) - menthol	1341.0 (43.0) - 3R4F<br>913.0 (30.0) -1R5F
N-nitrosoanabasine (NAB) (ng)	5.6 (0.8) - regular<br>2.6 (0.4) - menthol	65.0 (1.0) - 3R4F<br>46.0 (1.0) - 1R5F
Nicotine derived nitrosoamine ketone (NNK) (ng)	51.1 (0.3) - regular<br>58.2 (2.0) - menthol	532.0 (24.0) - 3R4F<br>412.0 (10.0) - 1R5F
Total of TSNA (ng)	250.8.1 (2.7) - regular<br>258.9 (4.0) - menthol	3826.0 (63.1) - 3R4F<br>3061.0 (92.0) - 1R5F

	**HTP aerosol**	**TCC smoke**
Total particulate matter (mg)	44.0 (11.4) - regular<br>49.9 (8.6) - menthol	36.9 (1.9) - 3R4F<br>28.9 (2.3) - 1R5F
Tar (mg)	9.8 (3.0) - regular<br>13.4 (2.2) - menthol	25.2 (1.5) - 3R4F<br>19.2 (1.3) - 1R5F
Nicotine (mg)	1.1 (0.1) - regular<br>1.2 (0.1) - menthol	1.7 (0.1) - 3R4F<br>1.0 (0.1) - 1R5F
Carbonmonoxide (mg)	0.4 (0.04) - regular<br>0.4 (0.04) - menthol	33.0 (1.8) - 3R4F<br>29.7 (1.7) - 1R5F
TSNAs (ng)		
NNN (ng)	19.2 (2.1) - regular<br>24.9 (3.5) - menthol	311.1 (24.3) - 3R4F<br>240.7 (6.6) - 1R5F
NAT (ng)	34.0 (3.1) - regular<br>37.2 (3.9) - menthol	246.4 (16.9) - 3R4F<br>183.1 (6.0) - 1R5F
NAB (ng)	4.5 (0.5) - regular<br>5.5 (0.6) - menthol	30.4 (2.0) - 3R4F<br>26.2 (0.5) - 1R5F
NNK (ng)	12.3 (1.5) - regular<br>13.8 (2.6) - menthol	250.4 (13.7) - 3R4F<br>107.0 (5.0) - 1R5F
Total of TSNA (ng)	70.0 (7.2) - regular<br>81.4 (10.4) - menthol	838.2 (53.7) - 3R4F<br>557.1 (15.7) - 1R5F

Supplementary table S2: Nicotine **c**oncentration present in heated tobacco product mainstream aerosol (HTP aerosol), mainstream smoke of traditional combustible cigarettes (TCC smoke), and mainstream electronic cigarette aerosol (ECIG aerosol). Health Canada Intense puffing regime was used. Reported HTP aerosol data was obtained from tobacco/regular/menthol flavoured HTP sticks. TCC smoke data was obtained from Marlboro Red 100 and Marlboro regular cigarettes. ECIG aerosol data was obtained from tobacco flavoured ECIG-liquid using 1st and 2nd generation ECIG devices. Values are mean (standard deviation). Sources: Leigh et al. 2018, Farsalinos et al. 2018 [2,3].

	**HTP aerosol (12 puffs) (Tobacco flavor)**	**TCC smoke (8 puffs) (Marlboro Red 100)**	**ECIG aerosol (55 puffs) (MarkTen, 3-5% nicotine, tobacco flavor)**
Nicotine (mg/cigarette)	1.4 (0.2)	2.1 (0.1)	1.3 (0.2)
	**HTP aerosol**	**TCC smoke** (Marlboro regular)	**ECIG aerosol** (2% nicotine)
Nicotine (mg/12 puffs)	1.40 (0.16) - regular<br>1.38 (0.11) - menthol	1.99 (0.20)	2 s puff:<br>0.46 (0.06) - 0.51 (0.05)<br>4 s puff:<br>0.86 (0.08) - 1.73 (0.20)<br>1st generation: cigaLike/ 2nd generation: eGO

Supplementary table S3: Comparative range of harmful and potentially harmful constituents (per cigarette) present in mainstream heated tobacco product mainstream aerosol (HTP aerosol) and mainstream smoke of traditional combustible cigarettes (TCC smoke). Blanks indicate not reported/not quantified/below level of detection. Single values are provided where range could not be obtained. ISO-International Organization for Standardization and HCI-Health Canada Intense regimes were used. Reported HTP aerosol data was obtained from tobacco flavoured HTP sticks, TCC smoke data was obtained from Marlboro Red cigarettes and reference grade (3R4F) cigarettes. Values are mean (standard deviation wherever available). Sources: Salman et al. 2019, Li et al. 2019 [4,5].

	**HTP aerosol**		**TCC smoke (Marlboro Red)**	
Smoking/Vaping regime	ISO	HCI	ISO	HCI
Total particulate matter (mg)	12.93 (0.25)	27.17 (2.08)	10.03 (0.65)	37.5 (2.96)
Nicotine (mg)	0.77 (0.06)	1.50 (0.2)	0.80 (0.05)	1.80 (0.11)
% Free Base	13.6 (1.4)	5.7 (2.2)	14.5 (1.9)	5.8 (1.7)
Propylene glycol/ glycerol	-	8/92	-	-
Reactive Oxygen Species nmol H_2O_2/cigarette				
Gas phase	1.93 (0.95)	2.25 (0-74)	22.10 (0.74)	-
Particle phase	4.34 (1.8)	7.78 (1.46)	24.74 (4.18)	-
Total	6.26 (2.72)	10.74 (2.12)	46.83 (9.6)	-
Carbonyls (µg)				
Formaldehyde	-	0.85 (0.28)	-	3.17 (0.33)
Acetaldehyde	-	301.46 (15.8)	-	1059 (9.03)
Acetone	-	48.37 (2.73)	-	775.6 (28.42)
Propionaldehyde	-	22.25 (0.6)	-	47.89 (1.04)
Crotonaldehyde	-	5.52 (0.55)	-	40.42 (0.69)
Methacrolein	-	6.53 (0.37)	-	85.46 (3.85)
Butyraldehyde	-	30.73 (1.89)	-	22.19 (2.91)
Valeraldehyde	-	20.11 (1.48)	-	-
Glyoxal	-	3.11 (0.18)	-	-
Methyl glyoxal	-	33.51 (1.23)	-	-
Sum of carbonyls	-	472.4 (19.35)	-	2033 (35.72)

	**HTP aerosol**		**TCC smoke** (3R4F)	
Smoking/Vaping regime	ISO	HCI	ISO	HCI
Total particulate matter (mg)	25.70 (0.84)	55.82 (1.10)	9.77	37.70
Nicotine (mg)	0.50 (0.03)	1.35 (0.07)	0.71	1.90
Tar (mg)	7.47 (0.42)	16.60 (0.42)	7.98	25.50
Propylene glycol (mg)	0.23 (0.01)	0.63 (0.05)	-	-
Glycerin (mg)	1.59 (0.02)	3.84 (0.12)	0.80	2.34
CO (mg)	0.25 (0.06)	0.52 (0.04)	11.20	32.70
Volatile organic compounds (µg)	1.54	6.77	579.20	1318.50
1,3-butadiene	-	0.45 (0.03)	38.50	76.50
Isoprene	0.58 (0.06)	3.02 (0.25)	395.00	863.00
Acrylonitrile	-	0.21 (0.01)	26.40	67.00

Benzene	0.12 (0.01)	0.61 (0.04)	45.70	104.00
Toluene	0.84 (0.05)	2.48 (0.18)	73.60	208.00
Carbonyls (µg)	191.27	308.24	948.80	2899.73
Formaldehyde	8.84 (0.43)	21.87 (0.81)	20.00	68.10
Acetaldehyde	128.50 (9.96)	210.00 (21.71)	567.00	1534.00
Acetone	18.83 (0.48)	26.59 (1.17)	210.00	690.00
Acrolein	4.01 (0.15)	6.37 (0.31)	56.70	155.00
Propionaldehyde	9.59 (0.19)	11.76 (0.38)	48.40	124.00
Crotonaldehyde	2.39 (0.09)	6.42 (0.28)	10.10	43.10
Butanal	14.87 (0.22)	18.77 (0.53)	25.60	65.03
2-Butanone	4.24 (0.46)	6.46 (0.33)	11.00	220.50
Aromatic Amines (ng)	-	-	19.36	38.12
1-Aminonaphthalene	-	-	10.62	21.60
2-Aminonaphthalene	-	-	5.69	10.10
3-Aminobiphenyl	-	-	2.04	4.18
4-Aminobiphenyl	-	-	1.01	2.24
Hydrogen Cyanide (µg)	-	-	70.90	319.00
Ammonia (µg)	2.41(0.44)	10.50 (1.62)	11.10	28.70
Tobacco specific nitrosamines (TSNAs) (ng)	17.20	41.50	280.10	794.00
N-nitrosonornicotine (NNN)	5.00 (0.32)	10.50 (0.46)	92.10	276.00
Nicotine derived nitrosoamine ketone (NNK)	3.50 (0.17)	7.30 (0.34)	85.50	243.00
N´-nitrosoanatabine (NAT)	6.10 (0.42)	18.10 (0.67)	92.90	251.00
N-nitrosoanabasine (NAB)	2.60 (0.15)	5.60 (0.31)	9.60	24.00
Phenol (µg)	-	1.20 (0.05)	7.04	14.80
Polycyclic aromatic hydrocarbon (ng) Benzo(a)pyrene	-	-	6.73	16.20

Supplementary table S4: Comparative range of harmful and potentially harmful constituents (per cigarette) present in heated tobacco product mainstream aerosol (HTP aerosol), mainstream smoke of traditional combustible cigarettes (TCC smoke) and mainstream electronic cigarette aerosol (ECIG aerosol). HCI- Health Canada Intense and CORESTA- Cooperation Centre for Scientific Research Relative to Tobacco puffing regimes were used. Reported HTP aerosol data was obtained from tobacco/regular and menthol flavoured HTP sticks. TCC smoke data was obtained from reference grade (1R6F) and Marlboro Red cigarettes. ECIG-aerosol data was obtained from tobacco flavoured ECIG-liquid using 1st and 2nd generation ECIG devices at 10 and 14 wattage. Values are mean (standard deviation wherever available). **PR:** puffing regime. Sources: Farsalinos et al. 2018, Bitzer et al. 2020 [6,7]

Puffing regime: HCI		**HTP aerosol**		**TCC smoke**	**ECIG aerosol (ECIG liquid: 18mg/ml nicotine; Evic VTC mini**	
PR1: 55 ml puff volume, 2 s puff duration, 3 s inter puff interval (12 puffs); PR2: 80 ml puff volume, 3 s puff duration, 30 s inter puff interval (12 puffs); PR3: 90 ml puff volume, 3 s puff duration, 25 s inter puff interval (14 puffs)						
	Puffing regime	Regular	Menthol	Marlboro red	ECIG power 10 watt	ECIG power 14 watt
Formaldehyde (µg)	PR1	6.4 (1.8)	5.0 (1.4)	67.2 (14.0)	0.5 (0.2)	1.0 (0.2)
	PR2	9.1 (3.7)	13.6 (5.3)	74.4 (24.2)	2.8 (0.9)	3.1 (0.4)
	PR3	17.1 (2.4)	22.6 (4.8)	71.4 (15.4)	3.6 (1.7)	3.7 (6.6)
Acetaldehyde (µg)	PR1	144.1 (23.3)	176.7 (32.6)	1062.2 (161.4)	0.8 (0.3)	1.5 (0.3)
	PR2	146.8 (22.3)	187.8 (61.8)	1372.9 (191.2)	1.9 (0.6)	1.9 (03.3)
	PR3	165.1 (10.5)	187.1 (13.4)	1212.6 (144.2)	1.7 (0.4)	2.9 (0.6)
Acrolein (µg)	PR1	10.8 (4.0)	10.4 (1.9)	112.7 (14.2)	0.3 (0.1)	0.4 (0.1)
	PR2	8.1 (2.0)	10.6 (6.1)	156.8 (32.1)	0.5 (0.3)	1.0 (0.1)
	PR3	10.4 (1.8)	13.1 (1.2)	160.9 (19.3)	0.9 (0.1)	1.1 (0.1)
Propionaldehyde (µg)	PR1	12.8 (3.7)	11.0 (2.4)	108.6 (17.9)	-	-
	PR2	8.8 (4.6)	13.2 (4.0)	113.7 (14.1)	-	-
	PR3	11.8 (1.5)	13.5 (1.3)	117.1 (15.4)	-	-
Crotonaldehyde (µg)	PR1	2.0 (0.4)	1.9 (0.2)	41.1 (14.6)	-	-
	PR2	1.4 (0.5)	2.4 (0.9)	65.7 (9.5)	-	-
	PR3	3.0 (0.7)	3.3 (0.6)	40.5 (8.6)	-	-

Table S4 continued

Puffing regime: CORESTA	HTP aerosol		TCC smoke	HTP aerosol (Hybrid)	ECIG aerosol		
Puff volume 75ml, puff duration: 2.5 s, inter puff interval: 30 s	IQOS<br>12 puffs	Glo<br>7 puffs	1R6F<br>11 puffs<br>(1 cigarette)	Ploom<br>10 puffs	JUUL<br>10puffs	SREC<br>10puffs	Mod<br>10 puffs
Nicotine (μg)	122.2 (9.6)	72.1 (10.6)	189.5 (7.9)	18.0 (0.0)	155.7 (44.6)	71.0 (8.2)	Nicotine free
Particulate phase radicals (pmol)	-	-	79.39 (7.5)	-	-	-	-
Total gas phase radicals (pmol)	12.6 (1.1)	12.5 (0.3)	567.6 (78.3)	12.1 (1.4)	5.3 (0.5)	39.6 (0.8)	47.8 (1.8)
Non-polar characteristics gas phase radicals (pmol)	13.9 (0.9) - 110%	14.3 (2.8) - 115%	449.9 (86.9) - 78%	11.3 (2.2) - 93%	2.4 (1.4) - 46%	14.4 (0.7) - 36%	19.2 (0.3) - 40%
Polar characteristics gas phase radicals (pmol)	6.8 (1.6) - 54%	8.2 (1.9) - 66%	9.6 (2.8) -2%	7.0 (1.0) - 58%	5.9 (1.1) - 113%	39.2 (0.9) - 99%	43.3 (2.9) - 91%

References

1 Bekki, K., Inaba, Y., Uchiyama, S. & Kunugita, N. Comparison of chemicals in mainstream smoke in HTP tobacco and combustion cigarettes. *Journal of UOEH* **39**, 201-207, doi:10.7888/juoeh.39.201 (2017).

2 Farsalinos, K. E., Yannovits, N., Sarri, T., Voudris, V. & Poulas, K. Nicotine delivery to the aerosol of a HTP tobacco product: Comparison with a tobacco cigarette and E-cigarettes. *Nicotine & tobacco research : official journal of the Society for Research on Nicotine and Tobacco* **20**, 1004-1009, doi:10.1093/ntr/ntx138 (2018).

3 Leigh, N. J., Palumbo, M. N., Marino, A. M., O'Connor, R. J. & Goniewicz, M. L. Tobacco-specific nitrosamines (TSNA) in heated tobacco product IQOS. *Tobacco control* **27**, s37-s38, doi:10.1136/tobaccocontrol-2018-054318 (2018).

4 Li, X. *et al.* Chemical analysis and simulated pyrolysis of tobacco heating system 2.2 compared to conventional cigarettes. *Nicotine & tobacco research : official journal of the Society for Research on Nicotine and Tobacco* **21**, 111-118, doi:10.1093/ntr/nty005 (2019).

5 Salman, R. *et al.* Free-base and total nicotine, reactive oxygen species, and carbonyl emissions from IQOS, a heated tobacco product. *Nicotine & tobacco research : official journal of the Society for Research on Nicotine and Tobacco* **21**, 1285-1288, doi:10.1093/ntr/nty235 (2019).

6 Bitzer, Z. T., Goel, R., Trushin, N., Muscat, J. & Richie, J. P., Jr. Free Radical Production and Characterization of HTP Cigarettes in Comparison to Conventional and Electronic Cigarettes. *Chemical research in toxicology* **33**, 1882-1887, doi:10.1021/acs.chemrestox.0c00088 (2020).

7 Farsalinos, K. E. *et al.* Carbonyl emissions from a novel heated tobacco product (IQOS): comparison with an e-cigarette and a tobacco cigarette. *Addiction (Abingdon, England)* **113**, 2099-2106, doi:10.1111/add.14365 (2018).
